# Cellular Ser/Thr-Kinase Assays Using Generic Peptide Substrates

**DOI:** 10.2174/1875397300801010054

**Published:** 2008-05-23

**Authors:** Deanna G Adams, Yu Wang, Puiying A Mak, Jason Chyba, Orzala Shalizi, Jason Matzen, Paul Anderson, Tim R Smith, Michael Garcia, Genevieve L Welch, Emmanuel J Claret, Michel Fink, Anthony P Orth, Jeremy S Caldwell, Achim Brinker

**Affiliations:** 1 Genomics Institute of the Novartis Research Foundation, San Diego, California, USA; 2 Cisbio International, HTRF/Bioassays, Bagnols-sure-Ceze, France

## Abstract

High-throughput cellular profiling has successfully stimulated early drug discovery pipelines by facilitating targeted as well as opportunistic lead finding, hit annotation and SAR analysis. While automation-friendly universal assay formats exist to address most established drug target classes like GPCRs, NHRs, ion channels or Tyr-kinases, no such cellular assay technology is currently enabling an equally broad and rapid interrogation of the Ser/Thr-kinase space. Here we present the foundation of an emerging cellular Ser/Thr-kinase platform that involves *a)* coexpression of targeted kinases with promiscuous peptide substrates and *b)* quantification of intracellular substrate phosphorylation by homogeneous TR-FRET. Proof-of-concept data is provided for cellular AKT, B-RAF and CamK2δ assays. Importantly, comparable activity profiles were found for well characterized B-Raf inhibitors in TR-FRET assays relying on either promiscuous peptide substrates or a MEK1(WT) protein substrate respectively. Moreover, IC_50_-values correlated strongly between cellular TR-FRET assays and a gold standard Ba/F3 proliferation assay for B-Raf activity. Finally, we expanded our initial assay panel by screening a kinase-focused cDNA library and identified starting points for >20 cellular Ser/Thr-kinase assays.

## INTRODUCTION

Over the last decades, cellular screening and profiling efforts have positively impacted early drug discovery pipelines from initial hit finding through lead optimization [1, 2]. The development of broadly applicable assay technologies, e.g. Ca^2+^-influx assays, cAMP quantitation and other secondary messenger assays for GPCRs or ion channels [3, 4], reporter gene assays for NHRs [5, 6] or Ba/F3 proliferation assays for tyrosine kinases [7, 8] have facilitated the systematic exploitation of traditional drug target families. New platform technologies continue to be developed to better suit the needs and economics of (u)HTS, to help tackle “difficult” and orphan targets or simply to provide orthogonal readout alternatives for hit reconfirmation purposes [9-12].

Recent advances in automated uHTS technologies have enabled the quantitative and parallel interrogation of arrays of cellular assays [8, 14]. In an earlier study, we reported on an automated large-scale experiment profiling 35 cellular Tyr-kinase assays against a focused inhibitor library [8]. Several repurposing opportunities for known drugs and possible opportunistic entry points for future drug discovery programs were identified. A similarly rich set of automation friendly assays is not currently available to interrogate the Ser/Thr-kinase space in an equally effective manner. Promising attempts have been made to quantify intracellular protein phosphorylation of selected Ser/Thr-kinase substrates in a HTS compatible format [9, 15, 16]. However, the generalization of these strategies is currently hindered by the limited availability of high-quality phospho-specific antibodies.

Here, we present the foundation of a cellular Ser/Thr-kinase assay platform that relies on promiscuous peptide substrates for the quantification of intracellular kinase activities. *In vitro,* a minimal reagent set of three peptide substrates in conjunction with one phospho-specific antibody [17] is sufficient for the development of >100 biochemical Ser/Thr-kinase assays (KinEASE^TM^-platform, Cisbio International). We have translated this biochemical platform into a cellular format while maintaining the original homogeneous addition-only protocol as well as the HTS-friendly TR-FRET readout [18-21]. In the case of B-Raf(V600E) targeted TR-FRET assays we found that a set of selected reference compounds returned comparable activity profiles for both protein and peptide substrate based cellular assays. Overexpressed target kinases like AKT and CamK2δ remained responsive to upstream activation pathways. Finally, through cDNA screening we were able to identify starting points for >20 cellular Ser/Thr-kinase assays covering diverse branches of the phylogenetic kinome tree. In general, cellular Ser/Thr-kinase assays were readily adaptable to high density screening in 384-well and 1536-well microtiter plates and are therefore considered well suited for future automated profiling campaigns.

## MATERIALS AND METHODS

### Materials

Europium-conjugated pSTK (61PSTKAY), pAkt (61P15 KAYC) pMEK1/2 (61P17KAYC), cMyc (61MYCKLB) antibodies and XL665-conjugated FLAG (61FG2XLB) and His6 (61HISXLB) antibodies were purchased from Cisbio, US. Unconjugated B-Raf antibody was from Santa Cruz Biotechnology (sc-5284), pMEK1/2 antibody from Cell Signaling Technology (9121), anti-FLAG M2 from Sigma. TR-FRET assays were performed in white tissue culture 384-well or 1536-well plates (Greiner Bio One). pcDNA5TO plasmid, HEK293 T-Rex cells, hygromycin, zeocin, and blasticidin were purchased from Invitrogen. Plasmid pSEMS1-26m was obtained from Covalys. Wortmannin and LY294002 were from Calbiochem and staurosporine from Sigma. Other reference compounds were synthesized at GNF.

### Generation of Substrate Constructs

Peptide substrate sequences were epitope-tagged and fused to AGT, *i.e.* the SNAP-tag [22-24]. Generic peptide substrates sequences were previously described [17]. For simplicity, SNAP-peptide constructs will be referred to as cSTKpep-1, 2 and 3. Complementary DNA oligonucleotides were synthesized (Valuegene) comprising the three peptide substrates with flanking Myc and FLAG epitope tags at the amino- and carboxy-terminus, respectively. Oligonucleotides were annealed and the resulting dsDNA ligated the into BamHI and NotI sites of pSEMS1-26m (Covalys). Full-length protein substrates like MEK1 were similarly epitope-tagged: A two-step PCR protocol using sense primer 5'TCAGAAGAGGATCTGATGCCCAAGAAGAAGCCGACG3' and antisense primer 5'GTCCTTGTAGTCGACGCCAGCAGCATGGGTTGG3', followed by a second PCR reaction using sense primer 5'GAACAACTCATCTCAGAAGAGGATCTGATG3', and antisense primer 5'TTACTTGTCATCGTCGTCCTTGTAGTCGAC3' to add myc (amino-terminus) and FLAG (carboxy-terminus) epitope-tags to full-length MEK1 cDNA (MGC clone ID: BC054754). The resulting second PCR product was cloned into pcDNA4 HisMax (Invitrogen). The kinase-inactive (K97M) and dominant-negative (S218/222A) constructs were generated by site-directed mutagenesis (QuikChange, Strategene) using the wild-type full-length MEK1 construct as template.

### Generation of Tetracycline Inducible Kinase Constructs, Single and Double Stable Cell Lines

Full-length B-Raf wild-type (WT) and B-Raf(V600E) (NM_004333) cDNAs were subcloned from pcDNA3.1 into pcDNA5TO (Invitrogen) using HindIII/ Not1 restriction enzyme sites. The kinase-inactive (KD) double mutant B-Raf (V600E, K483M) was generated by site-directed mutagenesis. Akt1 (NM_005163) cDNA was subcloned from pcDNA3.1 into pcDNA5TO (Invitrogen) using BamHI/XbaI restriction enzyme sites and a C-terminal His-tag was added to the amino-terminus by PCR. The kinase-inactive AKT-KD (K179M) point mutant was generated by site-directed mutagenesis. HEK293 T-Rex cells (Invitrogen) were cultured in DMEM supplemented with 10% fetal bovine serum (Hyclone) and 5μg/ml blasticidin. Stable pools of cells constitutively expressing either cSTKpeptide or MEK1 protein substrates were generated by transfection of cDNA plasmids and selection in G418 (1 mg/ml) or zeocin (200 μg/ml), respectively. Double stable cell lines expressing tetracycline-inducible kinases were subsequently generated by transfection of these “substrate-only” HEK293 T-Rex cells and selection in medium containing 175μg/ml hygromycin B.

### Cellular Ser/Thr-Kinase Assays by Transient Reverse Transfection (384-Well Plates)

HEK293 cells were transfected using Fugene 6 (Roche) at a density of 8000 cells/well in 384-well plates and a final transfection volume of 30μl. The amounts of kinase and substrate plasmids per well varied for each experiment and are specified in the figure legends. Cell lysis and detection of phosphorylated or total substrate levels was performed 48h post transfection by addition of 30μl detection buffer (50 mM Hepes pH 7.2, 0.1% BSA, 20mM EDTA, 0.8M KF, and 2% Triton) containing anti-FLAG XL665 (2ng/ul) and Eu-pSTK (0.18ng/μl) or Eu-pMEK1 (0.24ng/μl). For the relative determination of total substrate levels 30μl detection buffer was added per well containing anti-FLAG XL665 (2ng/μl) and Eu-cMyc (0.3ng/μl). Plates were incubated for 3-6h at room temperature and TR-FRET readings taken on Acquest, Analyst GT (Molecular Devices Corp.), Envision or Viewlux (Perkin Elmer) plate readers. Emission readings at 671nm and 618nm were expressed as a ratio and multiplied by a constant value of 100 to obtain “TR-FRET Ratios”.

### cDNA Screening

cDNA libraries and screening protocols were applied essentially as previously described [25, 26]. Briefly, a kinase focused library was assembled consisting of approximately 800 cDNAs encoding human or mouse derived serine/threonine and tyrosine kinases. Plasmids were arrayed into 384-well plates at 25ng per well. The transfection mixture was prepared by combining plasmid DNA for cSTKpep- 1, 2 or 3 constructs and Fugene-6, in serum-free DMEM (Invitrogen). After a 20min incubation at room temperature, 5μl of the transfection mixture was dispensed such that each well contained 3.125ng of the respective cSTKpep plasmid. Subsequently, 25μl cells were added to each well (8000/well) using μFill (Bio-Tek) dispensers. Cell lysis and plate reads were conducted 48h post transfection as described above. cDNA screens were performed in duplicates for each substrate construct. Prespotted known active kinases served as positive controls in each screen; “substrate-only” wells containing only cSTKpep plasmids served as negative controls. Kinase-induced substrate phosphorylation was expressed relative to substrate-only control wells and average fold-activation values were calculated from duplicates.

### Small Molecule Inhibition Studies

For LMW screening purposes in 384-well plates HEK293 T-Rex cells were either transiently transfected with tetracycline-inducible kinase and substrate constructs at a previously optimized ratio following the above mentioned reverse transfection protocol. Alternatively, double stable cells were plated directly at a density of 8,000cells/well in a volume of 30μl. 24h post plating, tetracycline (1μg/ml) was added to induce kinase expression for approximately 16h prior to compound addition. Kinase inhibitors were resuspended in DMSO and 12 point 1:3 serial dilutions were prepared. 100nl of compound stocks were pintool transferred into the assay plates resulting in a final DMSO concentration of 0.3%. After a 2h incubation period TR-FRET detection reagents detection reagents were added. For 1536-well plate screening cells were either transiently transfected in bulk or stable cells were seeded directly. For transient bulk transfections the transfection reagent mixture was prepared according to the following ratios: plasmid DNA: fugene 6: serum Free media = 1μg: 3μl: 21μl. For every 1ml of cells 1.25μg of kinase plasmid, 0.156μg of substrate plasmid (8:1), 4.22μl of Fugene 6 and 30μl of serum free media was prepared. The transfection mixture was then incubated at room temperature for 30 min and mixed into a HEK293 cell suspension (400 cells/μl). 2,000 cells/well were dispensed in 1536-well plates in 5μl media volumes and incubated at 37˚C. Kinase expression was induced in stable cells after 24h incubation through the addition of 1μl tetracycline (5μg/ml) followed by an additional 4-16 h incubation time before compound addition. Compound treatment in transient transfection experiments occurred 48h post seeding. In either case, 50nl DMSO stocks were pintool transferred into each well and incubated at 37˚C for a 2h period before addition of 4μl detection reagents. TR-FRET readings were taken after another incubation time of 4-24h. Z’ scores were determined as described [27].

### Immunoblot Analysis

HEK293 cells were harvested 48h post transfection in cell lysis buffer (20 mM Tris-HCl pH 7.5, 150 mM NaCl, 1 mM Na_2_EDTA, 1 mM EGTA, 1% Triton, 2.5mM sodium pyrophosphate, 1 mM Beta-glycerophosphate, 1 mM Na_3_VO_4_, 1ug/ml leupeptin, supplemented with 1X protease inhibitors (Sigma). Cellular extracts were resolved by SDS-PAGE (10% Bis-Tris Gel, Invitrogen), transferred to nitrocellulose membranes, and immunoblotted with the indicated antibodies. Immunolabeled proteins were visualized through Odyssey infrared imaging (Li-Cor, Lincoln, NE), with IRDye^TM^800 (Rockland Immunochemicals) and AlexaFluor® 680 (Molecular Probes)-conjugated fluorescent secondary antibodies.

### B-Raf(V600E)-Ba/F3 Proliferation Assay

The B-Raf(V600E)-Ba/F3 cell line was generously provided by Markus Warmuth (GNF). Ba/F3 cells were counted and diluted to a final concentration of 75,000 cells/ml in RPMI media and 50μl/well were plated into 384-well white solid bottom plates. 50nl of a 10mM compound stock in DMSO were pintool transferred followed by a 48h incubation time at 37°C for 48h. Finally, 25μl Cell-Titer-Glo (Promega) detection reagent was added each and luminescence read were taken on a CLIPR (Molecular Devices Corp.) reader.

## RESULTS AND DISCUSSION

### Translating Biochemical STK-Assays into Cells

**Fig. (1):**
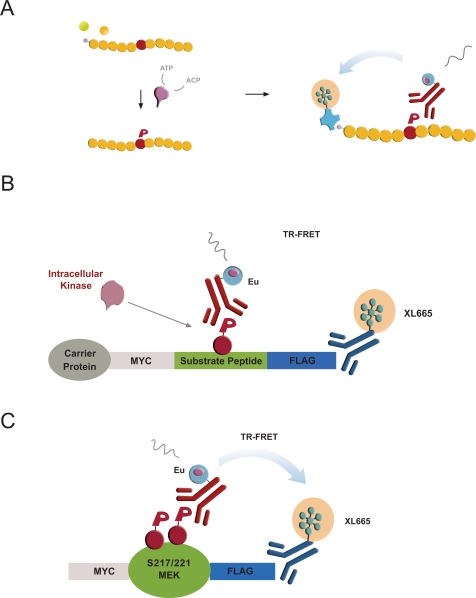
**Schematic of biochemical and cellular TR-FRET Ser/Thr-kinase assays.** (**A**) Biochemical KinEASE assay. (**B**) and (**C**) Cellular Ser/Thr-kinase assays. Target kinases are overexpressed in combination with cSTK peptide substrates (**B**) or full-length protein substrates like MEK1 (**C**). TR-FRET between phospho-specific and epitope-tag targeted antibodies is used to quantify intracellular substrate phosphorylation post lysis.

Cisbio’s KinEASE platform utilizes a minimal reagent set of three promiscuous peptide substrates (STK-peptides) sharing a common Ser-phosphorylation site and one corresponding phospho-specific antibody (pSTK) to support a panel of >100 biochemical Ser/Thr-kinase assays (Fig. **1A**, [17]). *In vitro, *the incubation of purified kinases with biotinylated peptide substrates leads to substrate phosphorylation quantifiable by TR-FRET [28]. We sought to translate this biochemical assay into a cellular format by first coexpressing Ser/Thr-kinases with reengineered cellular STK-peptide substrates –subsequently referred to as cSTK-pep– and then quantifying intracellular peptide phosphorylation using a simple homogeneous addition-only protocol.

To this end, we flanked the STK peptide sequences with N-terminal Myc- and C-terminal Flag-epitopes. The resulting oligopeptides were fused to hAGT carrier proteins (“SNAP-tag”) [22-24] to support their stable cellular expression and to have an option for a future direct functionalization of intracellular cSTKpep-1, 2 and 3 substrates (Fig. **1B**). The added epitope tags enable the quantification of either total substrate expression or substrate phosphorylation. To have a reference for peptide based cellular Ser/Thr-kinase assays we developed MEK1 as a model full-length protein substrate of kinases such as B-Raf or B-Raf(V600E), an activated oncogenic kinase mutant and established cancer drug target [31, 32].

### Case Study 1: B-Raf – Peptide *vs* Protein Substrates and Pharmacological Assay Validation

**Fig. (2). Case Study 1: B-Raf – Comparison of intracellular cSTKpep-3 vs. Mek1 protein substrates.**
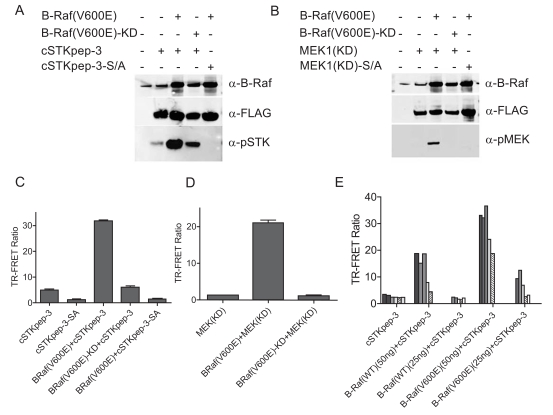
HEK293 cells were transiently double transfected (1µg kinase DNA and/or 1µg substrate DNA in 6-well dish) as indicated and immunoblotted. B-Raf constructs were combined with either (**A**) cSTKpep-3 or (**B**) kinase-inactive, MEK1(KD) protein substrates; (**C**)-(**E**) 384-well TR-FRET assays using a transient reverse transfection protocol. (**E**) B-Raf(WT) and B-Raf(V600E) constructs were cotransfected at 50ng or 25ng with a 2-fold serial dilution of cSTKpep-3 ranging from 25ng (black bar) to 1.5ng (white bar).

When the cSTKpep-3 substrate was expressed alone in HEK293 cells the observed basal phosphorylation levels derived from endogenous Ser/Thr-kinase activities were low, barely detectable by TR-FRET with weak phospho-specific bands being detectable by western blotting (Fig. **2A**, **C** and **E**). Similar observations were made with peptide substrates cSTKpep-1 and cSTKpep-2 (Fig. **8** and *data not shown*).

Cotransfection of cSTKpep-3 with B-Raf(V600E) resulted in a several fold increase in substrate phosphorylation by both western blotting and TR-FRET (Figs. **2A**, **C** and **E**). Moreover, B-Raf(V600E)-induced cSTKpep-3 phosphorylation was both dependent on the kinase activity as well as the presence of the target phosphorylation site since no cSTKpep-3 phosphorylation was detected when using a kinase-inactive mutant version of B-Raf(V600E), B-Raf(V600E)-KD or a Ser/Ala-mutant of cSTKpep-3 (cSTK pep-3-SA) (Fig. **2A** and **C**).

As expected, cSTKpep-3 phosphorylation could be modulated by both the amounts of substrate and kinase plasmids transfected as well as by the activation state of the transfected kinase (Fig. **2E**). Comparable results were obtained when using kinase-inactive, MEK1(KD), as a substrate (Figs. **2B** and **D**); cotransfection of MEK1 with B-Raf(V600E) resulted in increased MEK1 phosphorylation as detected by both western blotting and TR-FRET.

These results demonstrate that promiscuous Ser/Thr-kinase peptide substrates can be expressed in cells with low detectable levels of background phosphorylation and that significant increases in peptide substrate phosphorylation can be achieved through the overexpression of a target Ser/Thr-kinase. Since both the degree of background phosphorylation as well as kinase activities depend on the amounts of expression constructs applied in the transient transfection reactions, careful titrations of both constructs need to be performed to obtain optimal assay windows.

**Fig. (3):**
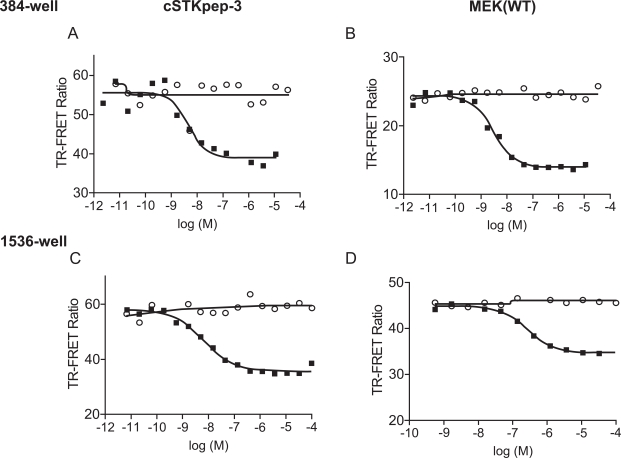
**Pharmacological validation of Raf(V600E)/cSTKpep-3 and Raf(V600E)/MEK(WT) TR-FRET assays. **IC_50_ curves were determined for B-Raf inhibitor A in 384-well and 1536-well plates using double stable cell lines. Open circles: DMSO, solid squares: B-Raf inhibitor A.

**Fig. (4):**
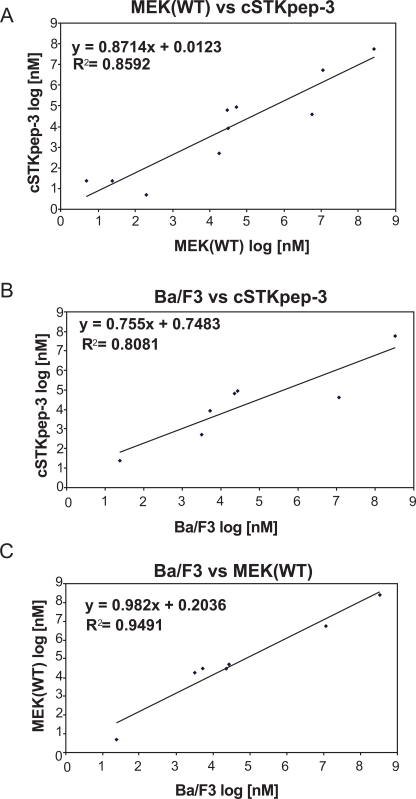
IC_50_ values of B-Raf inhibitors are strongly correlated between 384-well B-Raf(V600E) cSTKpep-3 and MEK1(WT) TR-FRET and Ba/F3 proliferation assays (Table **2**). (**A**) TR-FRET Raf(V600E)/cSTKpep-3 vs Raf(V600E)/MEK(WT). (**B**) TR-FRET Raf(V600E)/cSTKpep-3 vs B-Raf(V600E)-Ba/F3. (**C**) TR-FRET Raf(V600E)/MEK(WT) vs B-Raf(V600E)-Ba/F3.

For the pharmacological evaluation we generated double stable cells constitutively expressing either cSTKpep-3, wild-type MEK1(WT) or kinase-inactive MEK1(KD) substrates in combination with Tet-inducible B-Raf(V600E). Kinase inhibitors were then tested in 384-well or 1536-well formats. In all cases short compound treatment times of around 2h were employed to minimize cytotoxicity related compound interference (Figs. **3** and **4**, Table **1**).

Overall, cellular TR-FRET assays performed very robustly with B-Raf inhibitor A in both 384-well and 1536-well plates for MEK(WT), MEK(KD) or c-STKpep-3 substrates with Z’=0.6-0.9. Moreover, the observed potencies of a panel of selective in-house B-Raf inhibitors correlated very well in cSTKpep-3 and MEK1(WT) TR-FRET-assays (R^2^=0.86, Table **2**, Fig. **4A**). Good agreement was also observed when comparing TR-FRET assay results with historical Ba/F3-proliferation data for B-Raf(V600E) (R^2^_cSTKpep-3/BaF3_=0.81, R^2^_MEK1/BaF3_= 0.94, Table **1**, Fig. **4B** and **4C**). None of these B-Raf inhibitors affected basal cSTKpep-3 and MEK1(WT) background phosphorylation in the absence of prior B-Raf(V600E) induction (data not shown). Finally, a selection of unrelated kinase inhibitors, including Wortmannin and LY29002 did not show any activity in either the cSTKpep-3 or MEK1(WT) TR-FRET assays (Table **1**).

While B-Raf selective compounds compared very well in the above assays (Table **1**), pronounced discrepancies were reproducibly observed for the activities of the highly selective MEK1/2 inhibitor PD0325901 [34, 35] and staurosporine, a non-selective kinase inhibitor with only weak activity against Raf-kinases *in vitro*. Both compounds had comparable activities when using cSTKpep-3 or MEK1(WT) as B-Raf(V600E) substrates, but were inactive when using MEK1(KD). Similar observations of substrate dependent inhibitor profiles in cellular MEK-phosphorylation assays were recently made using an ELISA readout [36]. The underlying reasons for these differences are still being investigated. One possible explanation is the existence of a positive feedback loop driving ERK1/2 mediated (hyper)activation of B-Raf(V600E), although the detailed mechanism is still unclear and factors including the cellular context and predominant Raf isoforms involved may have an impact [29, 30]. According to this model, PD0325901 and staurosporine indirectly affect B-Raf(V600E) activity on cSTKpep-3 and MEK1(WT) by targeting downstream kinases and thereby antagonizing the activating feedback loop. Overexpression of MEK1(KD) on the other hand would exert the same effect as small molecule MEK1/2 inhibition, thereby rendering the residual B-Raf(V600E) activity insensitive to further PD0325901 inhibition. Regardless of the underlying mechanistic reasons for the observed discrepancies between B-Raf targeted TR-FRET assays, it is interesting to note the distinctions between the MEK1(WT) and MEK1(KD) assays. The nature and activation state of functional protein substrates can impact the cellular activities of upstream drug targets and the activity profiles of putative inhibitors. This is a potentially complicating factor that needs to be investigated when designing cellular enzyme assays around bioactive substrates [15, 16]. Biologically inactive peptide substrates on the other hand would be expected to have a comparatively minor effect on intracellular signaling pathways.

### Case Study 2: Interrogating the AKT Pathway and Probing for Compound Interference

**Fig. (5). Case Study 2: AKT dependent cSTKpep-3 phosphorylation.**
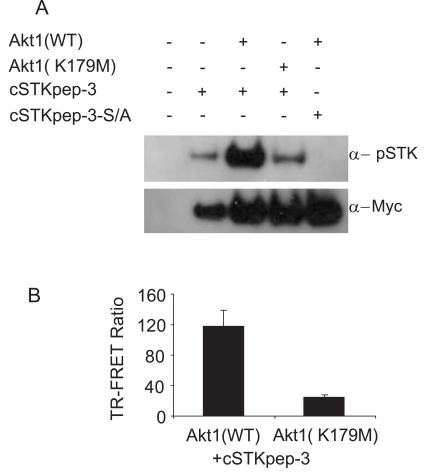
(**A**) HEK293 cells were transiently double transfected (1µg kinase DNA and/or 1µg substrate DNA in 6-well dish) as indicated and immunoblotted. (**B**) 384-well TR-FRET assay using a transient reverse transfection protocol. Wild-type Akt1(WT) or kinase-inactive Akt1(K179M) were combined with cSTKpep-3.

**Fig. (6). Quantification of total- and phospho-cSTKpep levels can reveal compound interference. The importance of short compound incubation.**
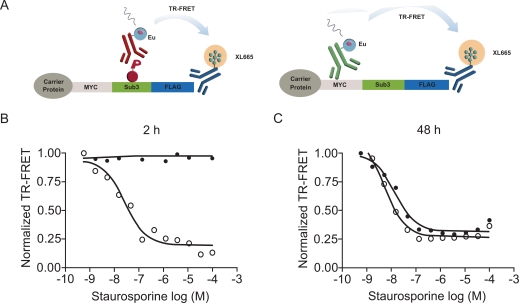
(**A**) Combinations of α-FLAG with α-p-STK or α-Myc antibodies were used to quantify either cSTKpep-phosphorylation or total cellular cSTKpep-3 expression by TR-FRET. (**B**) and (**C**) Transient 384-well AKT/cSTKpep-3 TR-FRET assay. Cells were treated with staurosporine for 2h (**B**) or 48h (**C**). Open circles: phospho-cSTKpep-3; Solid circles: total-cSTKpep-3.

For a second case study we turned to AKT and its upstream activation pathway. AKT-dependent intracellular cSTKpep-3 phosphorylation could be detected by western blotting and by TR-FRET (Fig. **5A** and **5B**). In the absence of other efficacious AKT inhibitors staurosporine was used as a tool compound and found to effectively inhibit AKT-dependent cSTKpep-3 phosphorylation (Fig. **6B**). Its cellular potency (IC_50_ ~100nM) agreed with the *in vitro* activity against AKT (IC_50_~100nM). Comparable IC_50_ values were found for incubation periods ranging from 30min (IC_50_~240nM) to 2h (IC_50_~100nM) (data not shown).

To determine if staurosporine-induced cytotoxicity and the subsequent loss of kinase substrate expression affected the outcome of the cellular assays, we aimed to separate the effect of staurosporine on cSTKpep-3 phosphorylation from its effect on total cSTKpep-3 expression (Fig. **6A**). After short 2h treatment staurosporine exclusively affected cSTKpep-3 phosphorylation, but not cSTKpep-3 expression levels. After an extended 48h treatment stauros-porine affected phospho- as well as total-cSTKpep-3 levels with equal apparent potency, suggesting that the occurrence of cytotoxicity inappropriately interfered with the assay readout.

These observations underline the importance of short compound treatment times to avoid high rates of uninteresting hits in an HTS context and further highlight the benefit of the presented cellular assay format over cell proliferation or reporter gene assays that typically require compound incubation times from several hours to days.

**Fig. (7). Targeting the AKT-pathway.**
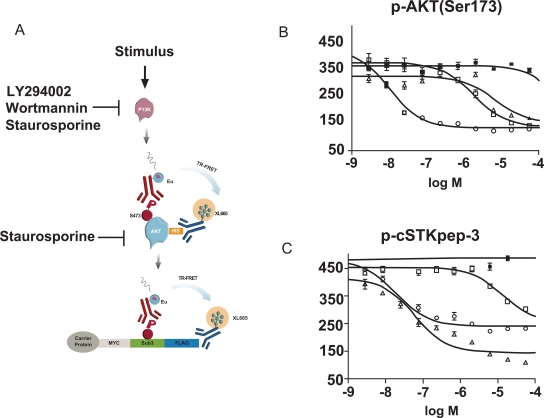
(**A**) TR-FRET assays were developed for the quantification of either p-AKT(Ser473) or p-cSTKpep-3. (**B**) p-AKT(Ser473) and (**C**) p-cSTKpep phosphorylation was quantified after treatment with Staurosporine (open triangles), Wortmannin (open squares), LY294002 (open circles) or DMSO (closed squares). Experiments were performed in 384-well plates after transient co-transfection of AKT-His and cSTKpep-3 into HEK293 cells.

Next, we aimed to interrogate the AKT pathway in greater detail by separating AKT activation as indicated by Ser473 phosphorylation, from signaling events depending on activated AKT such as cSTKpep-3 phosphorylation (Fig. **7A**). To this end we developed another TR-FRET assay using His-tagged AKT to monitor Ser473 phosphorylation.

As predicted for inhibitors of AKT activation, the PI3K inhibitors LY294002 and Wortmannin displayed comparable potencies in both AKT phosporylation (Fig. **7B**) and cSTKpep-3 phosphorylation (Fig.**7C**) assays. Moreover, the observed potencies of Wortmannin and LY294002 in the low nanomolar and low micromolar concentration ranges respectively were in line with their reported *in vitro* and cellular activities on PI3K [37, 38]. Staurosporine activity on the other hand differed by a factor of ~100fold when quantifying p-AKT (IC_50_~6μM) versus p-cSTKpep-3 (IC_50_~60nM). These cellular activities mirror the *in vitro* potency of staurosporine on PI3K (IC_50_~9μM) and AKT (IC_50_~100nM).

Together, these experiments demonstrate that the activity of overexpressed wild-type Ser/Thr-kinases can be responsive to upstream signaling events and that assays can be designed in the proposed format to interrogate a cellular pathway at different intersection points.

### Seeding a Cellular Ser/Thr-Kinase Assay Panel

Having shown proof-of-concept for two important drug targets AKT and B-Raf, we sought to identify additional Ser/Thr-kinases amenable to our cellular assay approach through cDNA screening. A kinase focused sublibrary of GNF’s genome scale cDNA collection [25, 26] comprising approximately 800 Ser/Thr- and Tyr-kinase clones was arrayed into microtiter plates. A panel of cSTKpep-1, 2 and 3 substrates was then screened besides a number of similarly designed SNAP-peptide constructs containing alternative substrate sequences, including Crosstide (GRPRTSSFAEPG), MBPtide (FFKKIVTPRTPPP), CREBtide (LRREIL SRRPSYRK) and the p38α-activation loop (LARHTDDEMTGYVA). cDNA hits resulting in >2fold S/N ratios relative to substrate-alone controls were picked for further activity and sequence confirmation. Between all 7 screens the three cSTKpep substrates combined scored ~86% of all hits (57/66) and overall higher S/N ratios than the other peptide substrates tested. cSTKpep-3 returned the majority of hits (37/57) followed by cSTKpep-2 (12/57) and c-Kinase-1 (8/57). Ultimately, 22 distinct Ser/Thr-kinases were identified as inducers of cSTKpep phosphorylation (Table **2**). Of these 22 Ser/Thr-kinases, 13 were already known members of the biochemical KinEASE-panel, whereas 9 kinases were newly identified. Nine out of the 13 previously known kinases preferred the same STK peptide substrate *in vitro* and in cells (Table **2**). No Tyr-kinases were found to stimulate cSTKpep phosphorylation, suggesting that no indirect Tyr-kinase induced activation of endogenous Ser/Thr-kinase pathways triggered cSTKpep phosphorylation. Overall, these observations are consistent with the idea that the intracellular kinase-cSTKpep interactions were direct.

### Future Outlook and Opportunities

**Fig. (8). Induction of CamK2δ activity.**
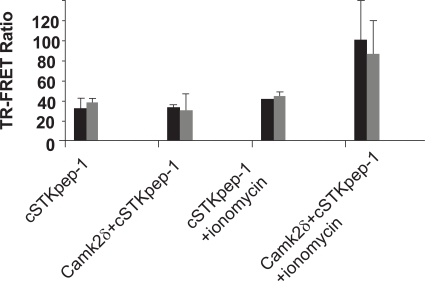
Transient transfection reactions were performed in 384-well plates as indicated. HEK 293 were reverse transfected with 50ng CamK2δ and 50ng (black bars) or 25ng (gray bars) cSTKpep-1. CamK2δ activity was induced through 5min ionomycin treatment (1µM) and p-cSTKpep-1 was quantified by TR-FRET.

While a number of kinases already represented on the biochemical KinEASE panel were picked up through DNA screening, others were missed. Several reasons might help explain these findings. Preactivation of Ser/Thr-kinases either through physiologic stimuli or *via *protein engineering, e.g. through the deletion of autoinhibitory domains or activating mutations, may be required beyond their plain overexpression to induce cSTKpep phosphorylation. The subcellular localization of kinases or their substrates, cell cycle dependency, cellular backgrounds and the dynamics of signaling pathways all may play a role. By rescreening our kinase cDNA library under modified assay conditions, e.g. in the presence of external stimuli, nuclear targeted substrates, different cell types, or alternatively through the design of an entirely new cDNA library of activated kinase constructs we aim to further expand the current panel of cellular Ser/Thr-kinase assays in the future. In the case of CamK2δ we found that transient activation of kinase activity and subsequent cSTKpep-1 phosphorylation was ionomycin inducible (Fig. **8**). We believe that assay conditions can be found to activate additional Ser/Thr-kinases in a similar manner.

## CONCLUSIONS

Here we present a starting point for the construction of a new cellular Ser/Thr-kinase assay platform that is tailored towards automated high density compound profiling and primary uHTS. We demonstrated that generic peptide substrates can form a viable basis for the determination of intracellular Ser/Thr-kinase activities and that a homogeneous TR-FRET protocol can be formulated to detect peptide phosphorylation in a sensitive, quantitative and automation-friendly manner. For B-Raf we showed that peptide- and protein-based cellular TR-FRET assays returned well correlated activity profiles for a panel of tool compounds ranging from highly selective to relatively promiscuous kinase inhibitors. Despite the overexpression of target kinases high-potency inhibitors could be readily identified as such. In addition, overexpressed target kinases like AKT and CamK2δ remained sensitive to upstream activating signals. Importantly, short compound incubation times on the order of 1-2 hours were sufficient to obtain robust IC_50_ values. This specific feature of the presented cellular TR-FRET kinase platform tremendously benefits HTS applications as it avoids artifactual cytotoxic hits or non-specific transcriptional inhibitors which tend to interfere with traditional proliferation or reporter gene assays of Ser/Thr-kinase activity. The builtin option to quantify relative substrate amounts as well as substrate phosphorylation by TR-FRET and thereby to normalize for substrate expression levels further helps to sort out non-specific compound interference. Finally, having validated cellular TR-FRET assays for two important drug targets, we expanded our assay concept to seed a panel of currently >20 cellular Ser/Thr-kinase assays through cDNA screening. While this initial panel will mainly target full-length wild-type kinases which are constitutively activated upon overexpression in HEK293 cells, a future generation of assays can be envisioned to include also engineered Ser/Thr-kinases or to involve kinase activation through physiologic stimuli. Wherever high-quality phospho-specific antibodies are available, protein kinase substrates will be incorporated into our panel as well. However, due to the very limited availability of such reagents it is expected that a rapid expansion and a significant coverage of the Ser/Thr-kinome will be initially achieved through TR-FRET assays relying on more broadly applicable peptide substrates like cSTKpep-1, 2 and -3 in conjunction with the corresponding universal p-STK antibody [17, 21]. In conclusion, the successful translation of the biochemical KinEASE assay platform into a cellular assay format will aid the exploration of biological Ser/Thr-kinase space through automated high-throughput screening and profiling.

## Figures and Tables

**Table 1:** **IC_50_ Profiles of B-Raf(V600E) TR-FRET and Ba/F3 Assays.** Standard deviations for data are listed in parentheses (n=4-6).

Inhibitor	IC50 [nM]			
	MEK(WT)	MEK(KD)	cSTKpep-3	Ba/F3
	384-well	384-well	384-well	384-well
Cmpd A	2(1)	1(1)	4(2)	4(3)
Cmpd B	112(31)	4(1)	140(77)	84(7)
Cmpd C	89(31)	21(8)	51(36)	41(3)
Cmpd D	4(2)	2(1)	4(3)	
Cmpd E	70(27)	19(1)	15(16)	33(22)
Cmpd F	1153(140)	7473(1615)	833(63)	
Cmpd G	846(601)	41(12)	100(2)	1160(394)
Cmpd H	10(5)		2(2)	
PD0325901	88(43)	NI	122(82)	78(35)
Bayer 43-9006	4542(1050)	376(179)	2309(625)	5000(800)
Wortmannin	NI	NI	NI	
LY294002	NI	NI	NI	
Cot inhibitor*	NI	NI	NI	
Staurosporine	117(56)	NI	106(40)	

* Cot Inhibitor: cmpd 30 [33]

**Table 2:** **An Emerging Cellular Ser/Thr-Kinase Assay Panel.** Confirmed hits obtained from cSTKpep cDNA screens. fold activation was calculated as TR-FRET signal with kinase and cSTKpep cotransfection divided by cSTKpep alone.

Gene Name	Accession number	Peptide Substrate		Fold Activation	Kinase Family
		Biochemical	Cellular		
B-Raf(V600E)	NM_004333		3	9.0	TKL
Cot	NM_005204		3	8.5	STE
PKA	BC054834	2	2	8.5	AGC
Akt1	NM_005163.1	3	3	9.9	AGC
Akt2	NM_001626	3	3	4.0	AGC
B-RAF(WT)	NM_004333		3	5.0	TKL
CHEK2	NM_007194.2	1	3	9.0	CMGC
MEKK3	NM_002401.3		3	8.9	STE
MLK3	NM_002419.2		3	5.3	TKL
Pim-2	BC018111	3	3	7.4	CAMK
PKD1	NM_002742.1		3	9.1	CAMK
PKD2	BC095949	1	3	5.9	CAMK
PRKX	NM_005044.1	2	2	9.0	AGC
Aurora-A	BC001280	2	2	4.3	AGC
BRSK2	NM_003957.1	1	3	2.2	CAMK
CAMK2*	BC032784	1	1	2.4	CAMK
MEK1-CA	NM_002755		3	3.4	STE
MKK3	BC032478		3	3.0	STE
MKK6	NM_002758.2		3	4.9	STE
NEK6	NM_014397.3	3	3	2.0	CAMK
NEK7	NM_133494.1	3	3	4.0	CAMK
PAK4	BC048238	2	3	2.9	STE

* ionmycin stimulation.

## ABBREVIATIONS

ACP: = automated cellular profiling
HEK: = human embryonic kidney
hAGT: = O^6^-alkylguanine-DNA-alkyltransferase
(u)HTS: = (ultra) high-throughput screening
KD: = kinase-inactive
LMW: = low molecular weight
MEK: = mitogen-activated protein kinase/extracellular signal-regulated kinase kinase
PI-3K: = phosphatidylinositol 3-kinase
Tet: = tetracycline
TR-FRET: = time-resolved fluorescence resonance energy transfer
WT: = wild-type
